# Interfacial film stabilized W/O/W nano multiple emulsions loaded with green tea and lotus extracts: systematic characterization of physicochemical properties and shelf-storage stability

**DOI:** 10.1186/1477-3155-12-20

**Published:** 2014-05-12

**Authors:** Tariq Mahmood, Naveed Akhtar, Sivakumar Manickam

**Affiliations:** 1 Department of Pharmacy, Faculty of Pharmacy and Alternative Medicine, The Islamia University of Bahawalpur, Bahawalpur 63100, Pakistan; 2 School of Pharmacy, The University of Faisalabad, Faisalabad 37610, Pakistan; 3 Manufacturing and Industrial Processes Research Division, Faculty of Engineering, University of Nottingham Malaysia Campus, Jalan Broga, Semenyih 43500, Malaysia

**Keywords:** Green tea, Lotus, Nano, Multiple, Emulsions, Stability

## Abstract

**Background and aims:**

Multiple emulsions have excellent encapsulating potential and this investigation has been aimed to encapsulate two different plant extracts as functional cosmetic agents in the W/O/W multiple emulsions and the resultant system’s long term stability has been determined in the presence of a thickener, hydroxypropyl methylcellulose (HPMC).

**Methods:**

Multiple W/O/W emulsions have been generated using cetyl dimethicone copolyol as lipophilic emulsifier and a blend of polyoxyethylene (20) cetyl ether and cetomacrogol 1000® as hydrophilic emulsifiers. The generated multiple emulsions have been characterized with conductivity, pH, microscopic analysis, phase separation and rheology for a period of 30 days. Moreover, long term shelf-storage stability has been tested to understand the shelf-life by keeping the generated multiple emulsion formulations at 25 ± 10°C and at 40 ± 10% relative humidity for a period of 12 months.

**Results:**

It has been observed that the hydrophilic emulsifiers and HPMC have considerably improved the stability of multiple emulsions for the followed period of 12 months at different storage conditions. These multiple emulsions have shown improved entrapment efficiencies concluded on the release rate of conductometric tracer entrapped in the inner aqueous phase of the multiple emulsions.

**Conclusion:**

Multiple emulsions have been found to be stable for a longer period of time with promising characteristics. Hence, stable multiple emulsions loaded with green tea and lotus extracts could be explored for their cosmetic benefits.

## Introduction

In these days the consumers worldwide are looking for personal care products, in specific the cosmetic products that assure multiple benefits. Besides, they also expect the latest advancements of technology to be incorporated into these innovative product formulations. Facing these trends, formulators strive to develop highly differentiated multifunctional product formulations that focus not only on the treatment but also on their aesthetics. A significant number of novel products are available in the markets that have been incorporated with a variety of new generation active ingredients. During the incorporation of these emerging actives, a range of formulation challenges are normally encountered that include control in the stability and the complications of combining several actives into a single cosmetic formulation [1]. Although, multiple emulsions were described in 1925; but much attention was not given until the report which was published in the late 1960s [2]. Recently, emulsions have established growing interest as vehicles to deliver the drugs efficiently to the body [3] and more importantly cavitation technique has been exploited to generate the nanoemulsions incorporated with the active constituents [4-7].

Complex water-in-oil-in-water (W/O/W) multiple emulsions consist of water-in-oil (W/O) dispersed into the continuous aqueous phase and stabilized with hydrophilic emulsifiers. Due to the unique structure and properties, these multiple emulsions are interesting carrier systems for various drug delivery approaches [8] especially for the controlled release of actives [9]. These multiple emulsions have been generated using cavitation technique [10-13]. In cosmetics, multiple emulsions play an important role to prepare skin care products with prolonged action [1].

However, compared with simple emulsions that consist of only two phases, much more destabilization processes need to be taken into consideration for the multiple emulsions. Four possible processes lead to the instability of W/O/W emulsions (a) coalescence of the internal aqueous droplets (b) coalescence of the oil droplets (c) rupture of the oil film resulting in the loss of the internal aqueous droplets, and (d) passage of water and water-soluble substances between the inner and outer aqueous phases. Thus, for the stabilization of multiple emulsions, following strategies have been proposed; (a) the use of high viscous oils to prevent the diffusion of water and water-soluble substances between the inner and outer aqueous phases, (b) the polymerization of interfacially adsorbed surfactant molecules, and (c) the gelation of oily or aqueous phases of the emulsions [14].

Green tea is among the most important plant extracts unfolded as cosmeceuticals [15] and it is now the subject of focus owing to its proven antioxidant properties and for its ability to repair photo-damage and phototoxicity caused by UV. It is also useful against a variety of skin disorders [16]. *Nelumbo nucifera* (lotus) extract has been observed to show potent antioxidant and anti-tyrosinase activities and has been found to have higher potential to be further developed as functional cosmetic agent [17,18].

The current study investigates the development of a stable cosmetic multiple emulsion loaded with green tea and lotus extracts which serve as functional cosmetic agents. Moreover, the influence of additives on the resultant aesthetic properties of these multiple emulsions have been investigated by subjecting the multiple emulsions for long term stability under varying conditions of temperature and humidity. Furthermore, it is the first time that a blend of hydrophilic emulsifiers, polyoxyethylene (20) cetyl ether and cetomacrogol 1000® has been employed in this study.

## Materials and methods

### Materials

To develop the multiple emulsions, paraffin oil was used as an oil phase (Merck, Germany). The lipophilic emulsifier Abil® EM 90 was supplied by Franken (Franken, Germany). Polyoxyethylene (20) cetyl ether (Brij 58® - Merck, Germany) and Cetomacrogol 1000® were used as hydrophilic emulsifiers. The thickener used was hydroxypropyl methylcellulose (HPMC, German Grade) and AnalaR grade MgSO_4_.7H_2_O (BDH, Poole, England) was used as a conductometric tracer. Standardized green tea and lotus extracts were used as functional cosmetic agents to be encapsulated in the inner phase of primary emulsion.

### Preparation of extracts

#### Extraction of green tea leaves

50 g of grounded and dried green tea leaves were first subjected to a continuous hot extraction process at 80°C for 6 h using a Soxhlet apparatus and by employing 90% ethanol. A second cycle of extraction with the above-stated conditions was applied for 12 h. A coarse filtration through muslin cloth was followed by a fine filtration (using Whatman Grade No. 1 filter paper). Filtrates were then concentrated in a rotary evaporator at 40°C and stored in a refrigerator till further usage.

#### Extraction of lotus plant

100 g of sacred lotus (whole plant material) was macerated using 2000 ml of 70% methanol overnight. The macerated mixture was then heated at 60°C along with continuous stirring using overhead blade mixer (Eurostar, Germany) at 1000 rpm for 2 h. The resultant mixture was then cooled down and coarse-filtration was carried out using several layers of muslin cloth. Fine filtration was then carried out using Whatman Grade No. 1 filter paper. Filtrates were then concentrated in a rotary evaporator at 40°C and then stored in a refrigerator till further usage.

The yield (%) obtained from both the extractions was calculated by using the following Equation 1,

(1) $$\mathrm{Yield}\;\left(\%\right)=100\;\left(W_{f}/W_{i}\right)$$

Where W_i_ is the initial weight of the plant material used for extraction and W_f_ is the weight of dried extract after solvent evaporation.

### DPPH radical scavenging activity

To measure the DPPH radical scavenging activity of green tea and lotus extracts, the method as reported by Lee and Shibamoto [19] was used. For this, 0.1 mM solution of DPPH was prepared in ethanol. Then 5 μL of the extract was dissolved in DMSO and mixed with ethanolic DPPH solution (95 μL). The above mixture was then dispersed in a 96-well microplates reader (Spectra Max plus 384 Molecular Device, USA) and incubated at 37°C for 30 min and the absorbance was measured at 515 nm. From this, the radical scavenging activity was determined by using the following Equation 2:

(2) $$\mathrm{DPPH}\;\mathrm{scavenging}\;\mathrm{effect}\;\left(\%\right)=1‒\left(\mathrm{Ac}–\mathrm{As}/\mathrm{Ac}\right)\;100$$

Where A_c_ = absorbance of control and A_s_ = absorbance of sample

### Preparation of multiple emulsions

Several pre-formulation studies were performed initially using different concentrations of oil and emulsifiers to arrive into a simple emulsion which was then further developed to fine multiple emulsions. Resultant simple emulsions with varying compositions of oil and emulsifier were stored at 40°C for a month and each sample was subjected to centrifugation and checked for stability at the end of one month. The most stable simple emulsion resistant to phase separation was then considered for the development of multiple emulsions. In the second stage of development of multiple emulsions, homogenization time and concentration of hydrophilic emulsifiers were varied to obtain a thicker creamy multiple emulsions. The concentration of HPMC was kept constant in all the experiments. The composition of multiple emulsion (MeC) formulations has been shown in Table 1.

**Table 1:** Composition of HPMC thickened green tea and lotus extracts loaded multiple emulsions (MeC) (% w/w)

**Primary emulsion (W/O)**	
Paraffin oil	24
Cetyl dimethicone copolyol	4.25
Green tea extract	2.5
Lotus extract	2.5
Magnesium sulfate	0.7
Deionized water (Q.S)	100
Multiple emulsion (W/O/W)	
Primary emulsion	80
Polyoxyethylene (20) cetyl ether	3.75
Cetomacrogol 1000®	2.5
Hydroxypropyl methylcellulose (HPMC)	1.25
Deionized water (Q.S)	100

All the multiple emulsions have been produced by a two-step emulsification strategy [20]. Briefly, simple emulsion was produced by emulsifying the oil with the lipophilic emulsifier and the mixture was preheated at 75°C before the emulsification. Green tea extract and conductometric tracer (MgSO_4_.7H_2_O) were then incorporated into the internal aqueous phase of the primary emulsion. Mixing of the aqueous phase with the oil phase continued at 2000 rpm for 15 min and then at 1000 rpm for 10 min. Finally the obtained emulsion was cooled down to room temperature while maintaining a mixing speed of 500 rpm for a further 10 min. Mixing was accomplished by IKA Mixing Overhead Stirrer (Eurostar, Germany). For the second stage emulsification, the simple emulsion was added to the aqueous phase containing hydrophilic emulsifier at room temperature. A stirring speed of 700 rpm was maintained until the formation of multiple emulsion which was confirmed by microscopic analysis.

### Stability studies

In this study the focus is on the emulsion stability and not on the release behavior as the emulsion is designed to be spread on the skin and rubbing will aid in the release of active compounds. For the stability studies, multiple emulsions were weighed (50 g) and packed in glass containers with 100 g capacity and were kept in the incubation chambers at different storage temperature for a period of 30 days (accelerated storage period). Different storage conditions that were applied: room temperature (25±1°C), low temperature (8±1°C), high temperature (40±1°C) and high temperature with humidity (40±1°C with 75% relative humidity). At the pre-determined intervals (24 h, 48 h, 7 d, 15 d and 30 d, 8 months and 12 months), samples were removed from the storage and allowed to reach to room temperature (25°C) prior to evaluating their physico-chemical characteristics.

### Characterization of multiple emulsions (MeC)

After the preparation of multiple emulsions (MeC) they were characterized by different physico-chemical parameters such as microscopic analysis, conductivity, pH, phase separation and rheology for a follow-up period of 30 days and the influence of different storage conditions on these parameters were also determined. Initially the formulation samples (MeC) were stored at 8°C, 25°C, 40°C and at 40°C with 75% relative humidity. Also, one sample was kept in a plastic container and the stability was followed for 12 months at 25 ± 10°C with 40 ± 10% relative humidity.

### Microscopic analysis

The microscopic analysis of the generated multiple emulsions were examined using an optical microscope (Nikon E200, Nikon, Japan) with a camera (DCM-35 USB 2.0 and Minisee Image software). Observations were made at 100 X magnification after diluting the multiple emulsions. Measurements of 100 droplets per sample per storage condition were performed. The obtained images were analyzed using the software Digimizer (Version 4.1.1.0, MedCalc Software, Mariakerke, Belgium). After calculating the droplet diameter, the coefficient of variation (CV) was calculated by using the following Equation 3:

(3) $$\begin{array}{c} \mathrm{CV}\;\left(\%\right)=\left[\mathrm{standard}\;\mathrm{deviation}\;\left(\mathrm{\mu m}\right)\right)\\ \;\left(/\mathrm{mean}\;\mathrm{droplet}\;\mathrm{diameter}\;\left(\mathrm{\mu m}\right)\right]\;100 \end{array}$$

### Conductometric analysis

Conductometric analysis of the undiluted multiple emulsion was performed to examine the release of the electrolyte that has been initially entrapped in the internal aqueous phase. The specific conductivity of the emulsions was directly measured by using a digital conductivity meter (WTW- Tetracon®, Germany) at 25±2°C. Conductivity tests were performed for the multiple emulsion formulations immediately after their preparation and after 24 h, 7 d, 15 d, 30 d, 8 months and 12 months that have been kept at different storage conditions.

### pH determination

The pH of fresh multiple emulsion formulations and the formulations kept at different storage conditions was determined by using a digital pH meter (ProfiLine pH 197, WTW, Germany). The pH measurements were also taken for the formulations after 24 h, 7 d, 15 d, 30 d, 8 months and 12 months.

### Centrifugation

The generated multiple emulsions were centrifuged at 25°C (12) (Hettich EBA 20, Germany) and at 5000 rpm for 20 min. Centrifugation of each formulation was performed after 24 h, 7 d, 15 d, 30 d, 8 months and 12 months that have been kept at different storage conditions.

### Rheological examination

The rheological properties and viscosity measurements of multiple emulsions were determined using a Brookfield programmable rheometer (Model DV.III; Brookfield engineering laboratories Inc. USA). Rheocalc V 3.2 (Microsoft Corporation) software was used as a supporting program during the measurements. 0.5 g of each of the formulation was weighed and the viscosities were determined at 25°C with spindle speeds ranging from 100 to 200 rpm by using a spindle CP 41.

### Thermal stress test with repeated centrifugation

After one month of testing under accelerated conditions, one sample was packed in a plastic container and monitored for 12 months at varying conditions of 25±10°C with 40±10% relative humidity. The above-mentioned conditions are very close to the change in the environmental conditions of Pakistan. The samples after 8 and 12 months were then subjected to three elevated temperatures i.e. 50°C, 60°C and 80°C for 30 min in a thermostated water bath (Model H-4, China). In the first sequence, three 1 ml samples taken in plastic tubes (Eppendorf) were heated at three different temperatures i.e. 50°C, 60°C and 80°C for 30 min, and at the end they were centrifuged at 5000 rpm for 20 min and observed for the presence of any phase separation. In the second sequence, one sample filled in the tube was subjected to 50°C for 30 min and centrifuged again to observe the presence of any phase separation. The sample that was subjected to 50°C confirmed the absence of separation which was then subjected to the next elevated temperature i.e. 60°C and centrifuged to detect any phase separation. It showed no phase separation at 60°C, which was then finally subjected to 80°C to detect any phase separation. After each stage of heating and centrifugation, samples were observed under the microscope to confirm the integrity of the globules of multiple emulsions. Before subjecting to thermal stress testing, pH and conductivity measurements were performed for the samples of 8 and 12 months to investigate the effect of varying environmental conditions on the above parameters.

## Results and discussion

### Antioxidant activity

The extraction yield was first determined on the basis of dry plant material used for extraction and then the antioxidant potential of both the plant extracts was determined. From the results, a stronger antioxidant activity has been found out from the green tea extract while lotus extract has shown a less potent antioxidant activity as compared to green tea extract. Previous studies confirmed that the antioxidant activity of crude extract of green tea is higher than that of the standard antioxidant, ascorbic acid [21]. Also, earlier reports established that the methanolic extract of lotus has lower activity as compared to BHA and ascorbic acid [22]. Similar to above studies, the green tea in our study has shown a stronger antioxidant activity than lotus, irrespective of the extraction yield of green tea that has been found to be much lower than lotus extract. Table 2 shows the antioxidant activities and the extraction yield obtained from these two extracts.

**Table 2:** Antioxidant activities and the extraction yield of green tea and lotus extracts

**Extract**	**DPPH radical scavenging activity (%)**	**Yield (%)**
Green tea	88 ± 0.2	2.1
Lotus	75 ± 0.41	4.15

### Microscopic analysis and centrifugation

Microscopic analysis is a useful and informative tool to understand the characteristics of multiple emulsions. Further, droplet size measurements indicate the stability of multiple emulsions as a faster increase in the droplet size with time is the indicator of lower stability of the system [23]. The characteristics of multiple emulsions were investigated at the formulation stage as well as for the followed 30 days at different conditions of storage i.e. 8°C, 25°C, 40°C and at 40°C with 75% relative humidity. Furthermore, multiple emulsions were subjected to extensive centrifugation at 5000 rpm for 20 min to accelerate the phase separation of the system for the followed-up period of 12 months to understand the stability.

**Figure 1:**
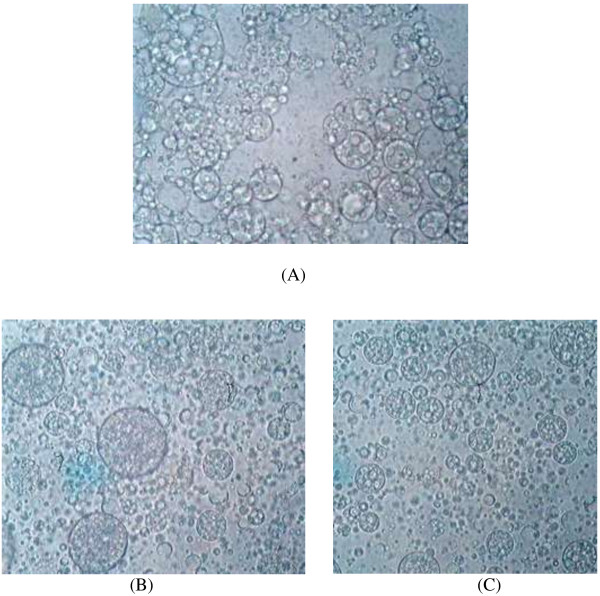
**Photomicrographs of multiple emulsion formulations kept at different storage conditions for 12 months. A** = Fresh sample, **B** = After 8 months, **C** = After 12 months.

No phase separation was observed in any of the samples upon extensive centrifugation. Mean globule size was in the range of 8.41±3 μm to 10.81±7 μm (mean ± standard deviation). The sample that was kept at 8°C has shown smaller size of globules while an increase in the globule size was observed with an increase in the storage temperature. A maximum increase in the globule size was observed at 40°C. However this increase in the globule size with temperature does not seem to be a significant as the coefficient of variation (CV) was only varying in a narrow range at all the studied storage temperatures. A larger CV indicates an unacceptable limit of shrinkage or coalescence of the globules which indicate a lack of uniformity of globules. As reported earlier, an increase in the globule size is due to coalescence phenomena, while a decrease in the size is due to leakage of water from the internal to the external aqueous phase [24]. Results from the microscopic and centrifugation stability studies have been shown in Tables 3, 4 and 5. Photomicrographs of multiple emulsion formulations that have been kept at different storage conditions have been shown in Figure 1.

**Table 3:** Mean globule size and coefficient of variation (CV) of multiple emulsions followed for 30 days

**Conditions**	**Multiple droplets (μm)**	**Inner droplets (nm)**	**CV (%)**
8°C	8.05 ± 4.2	800 ± 20	0.53
25°C	8.92 ± 4.2	1200 ± 60	0.47
40°C	10.81 ± 7.0	1400 ± 50	0.65
40°C with 75% RH	10.50 ± 5.5	1400 ± 80	0.53

**Table 4:** Conductivity, pH and centrifugation stability of multiple emulsions kept at different storage conditions for 30 days

	**8°C**	**25°C**	**40°C**	**40°C with 75% RH**
		pH*		
24 h	5.00 ± 0.09	5.19 ± 0.20	4.99 ± 0.18	4.99 ± 0.07
7 d	4.97 ± 0.14	5.14 ± 0.07	4.91 ± 0.09	4.95 ± 0.10
15 d	4.92 ± 0.15	5.12 ± 0.02	4.90 ± 0.10	4.93 ± 0.07
30 d	5.35 ± 0.06	5.05 ± 0.04	5.12 ± 0.12	4.99 ± 0.12
		Conductivity**		
24 h	76.40 ± 1.90	76.37 ± 1.19	66.50 ± 1.80	63.53 ± 1.51
7 d	83.30 ± 1.37	85.90 ± 3.90	77.50 ± 1.71	69.83 ± 3.00
15 d	84.27 ± 1.32	89.87 ± 1.90	79.20 ± 3.30	74.20 ± 2.08
30 d	89.17 ± 2.80	57.73 ± 2.14	93.00 ± 1.20	60.30 ± 2.39
		Centrifugation stability***		
24 h	S	S	S	S
7 d	S	S	S	S
15 d	S	S	S	S
30 d	S	S	S	S

*pH (Initial) = 5.04 ± 0.05, **Conductivity (Initial) = 77 ± 1.05 (μS/cm), ***Centrifugation at 5000 rpm for 20 min for each sample, S = Stable.

**Table 5:** Physicochemical characteristics of multiple emulsions shelf-stored at 8 and 12 months and subjected to thermal stress test with and without repeated centrifugation

	**50°C**	**60°C**	**80°C**
**After 8 months**			
*Centrifugation stability			
Samples without repeated heating	S	S	S
Sample with repeated heating	S	S	S
Microscopic globule integrity			
Samples without repeated heating	S	S	S
Sample with repeated heating	S	S	S
**After 12 months**			
*Centrifugation stability			
Samples without repeated heating	S	S	S
Sample with repeated heating	S	S	S
Microscopic globule integrity			
Samples without repeated heating	S	S	S
Sample with repeated heating	S	S	S

*Centrifugation at 5000 rpm for 20 min for each sample, S = Stable.

### Conductometric and pH analysis

Incorporating a conductometric tracer in the inner aqueous phase of the primary emulsion is necessary to detect any leakage from the internal to the external aqueous phase of W/O/W emulsion. Measurement of conductivity also provides the information about the entrapment of active substances in the primary emulsion. The amount of release is directly proportional to the amount of active substance that is available in the external aqueous phase and a rapid release therefore does not favor the slow release of the active substance. No significant variation in the conductivity was observed over 30 days of storage period and at any of the temperature that was subjected to. These results reveal that conductometric tracer has not diffused or rupturing of oil layer did not occur over the followed period of 30 days. In general it is believed that during the storage conductivity increases due to i) diffusion of an electrolyte, ii) the coalescence of globules iii) destruction of oil film because of the osmotic pressure and the leakage of internal aqueous phase [25].

Conductivity values for the first 30 days decreased for the formulation that was kept at 40°C with 75% RH relative to the conductivity values of the fresh sample. This may be due to the formation of larger droplet size or phase separation. But no phase separation was observed at this condition. However, an increase in the droplet size was observed. A similar justification has been offered previously [26] where the reduction in the observed conductivities has been attributed to phase separation and larger droplet size. After 8 and 12 months of storage, conductivity values (51.7 ± 3.06, 29.3 ± 0.30 respectively) tend to decrease but yet no phase separation occurred even after heating the samples at 80°C. Normal pH of skin is in between 5 and 6, and 5.5 is considered to be the average pH of skin [27]. Results of our pH analysis indicate that there is no variation in the pH of multiple emulsions kept at different conditions of storage and even after 8 and 12 months of storage (5.4 ± 0.01, 5.6 ± 0.01 respectively) under varying temperature and humidity conditions. Results of this study (Table 4) indicate that different storage conditions did not have any influence on the pH and conductivity.

### Thermal stress test with repeated centrifugation

Thermal stress test with repeated centrifugation has been applied on multiple emulsions to study the stability of these emulsions under extensive stress conditions of temperature change and centrifugal force. The obtained results of this test have been shown in Table 5.

Obviously the generated multiple emulsions found to be extremely stable against elevated temperatures and the thermal stress could not produce any change in the globule integrity, phase inversion or phase separation. Even when the multiple emulsion was subjected to elevated temperatures and repeated centrifugation at 5000 rpm for 20 min, no phase separation was observed and globules have been found to be intact as observed through microscopic examinations. Hence, when the emulsion was tested after 12 months of storage at 25 ± 10°C with 40 ± 10% relative humidity it was found to be very stable and expected not to deteriorate soon. This resistance to phase separation appears due to the addition of Cetomacrogol 1000®, which acts as a film stabilizer and this has not been reported so far. The possible mechanism behind Cetomacrogol 1000® as film stabilizer is probably due to its self-bodying action in which rheological properties of the emulsions are related to gel networks formed in the continuous phases. This phenomenon has been presented previously when mixtures of emulsifiers of the surfactant-fatty alcohol type are used to stabilize oil in water emulsions [28,29].

### Rheological analysis

Consistency of cosmetic formulations is a very important aspect and is determined by the rheological methods. Varying shear rates were applied to multiple emulsions to determine the flow parameters, while fitting the data in power law equation 4:

(4) $$\tau =K\;\gamma^{n}$$

Where τ is the shear stress, γ is the shear rate, K is the consistency index and n is the flow behavior index.

**Figure 2:**
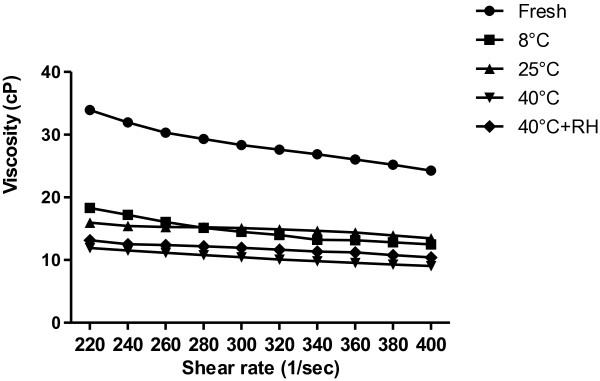
Viscosities of fresh sample and the samples kept at different conditions of storage for 30 days.

Consistency index K is a measure of the system consistency and it is related to apparent viscosity. Flow behavior index n determines the degree of non-newtonian flow behavior and varies in the range between 0 and 1. The non–newtonian behavior of the investigated system is more pronounced for smaller values of “n”. We measured the viscosities of multiple emulsions at a speed of 100 to 200 rpm while applying the shear rates from 200 to 400 on each of the samples. Varying shear rates were applied for the quality assurance of emulsions under stress conditions. Results for the rheology of fresh sample and for the samples that have been kept at different conditions of storage (followed for 30 days) have been shown in Figure 2.

Samples of multiple emulsions revealed non-newtonian flow and shear thinning behavior with different conditions of storage upon varying the shear rate. With an increase in shear rate and shear stress, a decrease in viscosity could be observed. Rheological analysis revealed excellent fits and were found to be from 98.6 to 99.6 and the obtained values of K and n have been shown in Table 6. It could be seen that all the samples exhibited pseudo-plastic behavior with the flow behavior index (n) between 0.40 and 0.78. At higher temperatures, samples exhibited low consistency and thus a moderate shifting towards newtonian behavior.

**Table 6:** Rheological analysis followed for 30 days

	**Initial**	**8°C**	**25°C**	**40°C**	40°C RH*
Consistency index (cP)	454.4	435.3	119.3	52.5	75.1
Flow index	0.51	0.40	0.57	0.78	0.67
Confidence of fit (%)	99.3	98.6	99.6	98.6	99

RH* = Relative humidity.

## Conclusion

Fascination towards novel topical formulations loaded with functional actives having better antioxidant activity have emerged in the recent era. Encapsulation has been carried out with plant extracts (5%) in W/O/W multiple emulsion that have been fabricated using different emulsifiers. Promising stability characteristics have been observed in the generated multiple emulsions that were kept under different storage conditions. More importantly, multiple emulsions kept at different storage conditions were stable toward any phase separation followed for the 12 months i.e. accelerated and under shelf-storage. Globule size remained constant at different storage conditions. In addition, the coefficient of variation was lower enough to speculate that there was no internal deformation in the multiple emulsion characteristics. Conductivity analysis revealed that entrapment efficiency of multiple emulsions was excellent enough to offer sustained release of bio-functional agents. Rheological analysis revealed excellent curve fittings for all the formulations and the formulations subjected to elevated temperatures showed shifting toward newtonian behavior. Based on the above observations, it is expected that the green tea and lotus extracts loaded multiple emulsions could be excellent carrier of these powerful antioxidant substances ensuring long term stability of actives.

## Competing interests

The authors declare that they have no competing interests.

## Authors’ contributions

All the authors have contribution in the design, planning and carrying out the experiments as well as drafting the manuscript. All the authors have read and approved the final manuscript.
